# MONTELUKAST DOES NOT INHIBIT THE LATE PHASE REACTION IN PARTHENIUM DERMATITIS

**DOI:** 10.4103/0019-5154.49006

**Published:** 2009

**Authors:** Lakshmi Chembolli, C R Srinivas

**Affiliations:** *From the Dept. of Dermatology, PSG Hospitals and PSGIMSR, Peelamedu, Coimbatore, Tamil Nadu, India. E-mail: srini_cr_1955@rediffmail.com*

Sir,

It has recently been postulated that Parthenium dermatitis is caused due to combined Type IV and Type I hypersensitivity.[1] Type I hypersensitivity, mediated by IgE, is characterized by an immediate wheal and flare at 15 minutes and a nodule - late phase reaction (LPR) at 24-48 hours.[1] The LPR is mediated by newly formed mast cell mediators (leukotrienes, prostaglandins, cytokines), in concert with inflammatory cells and is thought to be responsible for the chronic skin and bronchial hypersensitivity in atopic patients with dermatitis or bronchial asthma, respectively.[2]

Inhibition of leukotrienes plays an important role in the treatment of asthma and other allergic conditions such as allergic rhinitis, atopic dermatitis, and chronic urticaria. [3] Cysteinyl leukotrienes (CysLTs - LTC_4_, LTD_4_, LTE_4_) are potent proinflammatory mediators derived from arachidonic acid, through the 5-lipoxygenase pathway. They induce bronchoconstriction, increase vascular permeability and mucus secretion and facilitate the recruitment of eosinophils in the airways. They are the most potent bronchoconstrictors known to date. However, while the effects of CysLTs on the airways has been extensively investigated, their role in the pathogenesis of atopic dermatitis is still incompletely understood.[4] Leukotriene receptor antagonists (montelukast) antagonize the effect of CysLTs.

We studied the effect of montelukast on the LPR in an atopic patient with parthenium dermatitis (Serum IgE - 3731U/ml). The dermatitis was adequately controlled with azathioprine 50 mg daily, although the patient complained of occasional episodes of severe itching. Azathioprine is a synthetic purine analogue, which blocks purine synthesis. It exerts immunosuppressive effect by affecting the function of T and B cells. It is not thought to have an action on the release of histamine by mast cells. Since the patient was already on azathioprine 50 mg before and two months after the initiation of montelukast, it does not appear to have an action on the LPR. Subsequently, montelukast was administered along with rupatidine and colchicine in an HIV positive patient with parthenium dermatitis and failed to suppress the LPR.[5]

Montelukast 10 mg daily was administered along with azathioprine 50 mg. The immediate and late phase reaction following prick testing with parthenium leaf was recorded before and two months after the initiation of montelukast [Table 1].

**Table 1:** Results of prick testing with parthenium leaf

Reaction	Before montelukast	2 months after montelukast
Immediate reaction	5 mm	10 mm
LPR	5 mm	6 mm

Both the immediate reaction and the LPR were not controlled with montelukast. A few studies have reported the beneficial effect of montelukast in atopic dermatitis.[4] Another study has not confirmed sustained benefit in extensive atopic dermatitis,[6] although it is claimed to have shown significant benefit in the treatment of bronchial asthma and allergic rhinitis.[3,7,8] There are conflicting reports in the treatment of chronic idiopathic urticaria.[9,10] Since the immediate reaction had increased from 5 to 10 mm, a proportionate increase in the LPR is also expected.[11]

Montelukast may not be as effective in controlling the LPR in the skin as it is in the bronchial mucosa. Further studies are required to confirm the ineffectiveness of montelukast in suppressing the LPR in skin.
